# A verbal descriptor incremental pain scale developed by South African Tswana-speaking patients with low back pain

**DOI:** 10.4102/sajp.v74i1.460

**Published:** 2018-08-30

**Authors:** Michelle Yazbek, Aimee V. Stewart, Alison Bentley

**Affiliations:** 1 Department of Physiotherapy, University of the Witwatersrand, South Africa; 2 Department of Family Medicine, University of the Witwatersrand, South Africa

## Abstract

**Background:**

Measuring pain in patients whose home language is not English can be difficult as there may not be a scale available in their home language. Scales devised in other countries may also not be accurate after translation.

**Objectives:**

The aim of this study was to develop and test a new verbal pain descriptor scale in a Tswana-speaking population in South Africa with low back pain.

**Method:**

Two separate Tswana-speaking groups (20 males and 20 females) of patients with low back pain were asked to describe each of four categories of pain: mild, moderate, severe and worst. They then voted and descriptions obtaining more than 70% of the vote were taken to the next round of voting with both groups together. A final scale of one description for each category of pain (Tswana Verbal Pain Descriptor Scale – TVPDS) for both males and females was tested on a sample of 250 patients with low back pain and against three other non-verbal pain scales.

**Results:**

All items on the final scale were approved by at least 70% of both male and female participants. The scores for the TVPDS correlated well with present pain perception (*r* = 0.729, *p* < 0.0001) measured on the numerical visual analogue scale. The TVPDS correlated well with the Wong–Baker FACES Pain Rating Scale (*r* = 0.695, *p* < 0.0001) and the Pakistani Coin Pain Scale (*r* = 0.717, *p* < 0.0001).

**Conclusion:**

The TVPDS has the potential to be a useful clinical scale but more testing in other languages is still required.

**Clinical implications:**

This pain scale has the potential to be a useful scale to use for Tswana-speaking persons with low back pain and could also be useful for persons of other languages, if translated.

## Introduction

Whenever health care practitioners are required to assess and manage low back pain (LBP) they rely on patient descriptions of pain to judge the severity of the expressed pain. The expression of pain is dependent on language and people of different cultures and languages express increasing severity of pain in ways that are meaningful for them (Callister 2003). This is a particularly important issue if the health care practitioner and patient speak different languages, making the expression of pain difficult for the health care practitioner to understand.

This difficulty is frequently seen in a country like South Africa that has 11 official languages. This may lead to tension and misunderstanding between a health care practitioner from a different language needing to understand pain severity and the patient being unable to relate to a given pain scale or, as often happens, simply being asked to describe their pain (Callister 2003). A Zulu-speaking patient describing the pain of peripheral neuropathy as ‘cramping’, which is not the word a health care practitioner might expect, is an example of the difficulty patients and health care providers may have in understanding each other (Shaikh, Bentley & Kamerman 2013).

Numerous pain rating scales have been developed, both verbal and non-verbal, given the subjective nature of pain and the difficulty most people have in describing their pain (Gentile et al. 2011; Wong & Baker 1988). Most of these scales have been developed in Western societies and few, if any, take into consideration the differing language usage of patients. The verbal scales usually require knowledge of English or else are translated into local languages without considering the specifics of local language usage.

Most pain scales describe pain severity in categories of increasing pain intensity, examples being ‘mild, moderate, severe and worst’ (Collins, Moore & McQuay 1997; Jensen et al. 2007; Jones et al. 2007). This may be difficult for someone whose first language is not English and whose language may not have the same nuances to describe increases in pain severity. Asking people to describe the severity of their pain in their first language may be useful (Schott 2004; Semino 2010; Stewart 2014). Pain can be described using words, metaphors or verbal phrases. The use of everyday phrases to describe pain is common, where people try to match their pain to well-known situations in which pain occurs (Schott 2004; Semino 2010; Stewart 2014).

The first author, a practising physiotherapist in an area where most of her patients are Tswana speaking (one of the official South African languages), found that her patients did not understand the visual analogue scale (VAS), commonly used in clinical practice, when asked to rate their LBP. This left her unable to evaluate the level of pain being experienced. Her patients tended to be unsure of where on the VAS they should ‘place’ their perception of their pain (Yazbek, Stewart & Becker 2009). She therefore felt that a pain scale through which patients could describe their pain in their first language might be worth considering.

So this study aimed to develop a pain scale using everyday Tswana descriptions for increasing severity of pain and to test the relationship of this scale with existing pain scales.

## Method

This cross-sectional study was divided into two parts: Part 1, the development of the Tswana Verbal Pain Descriptor Scale (TVPDS), and Part 2, testing the TVPDS in a mixed group of Tswana-speaking individuals with LBP.

### Part 1: Development of the scale

Forty adult participants (older than 18 years) with LBP attending a spinal clinic at a provincial public hospital in South Africa were asked to participate in Part 1. The study process was explained to them in Tswana and they signed informed consent. All participants had chronic LBP because of various causes and were only excluded if they had other diseases that could cause pain. The specific causes of back pain were not considered important in this study as it was the participants’ ability to verbalise their pain that was of interest. There were 20 males and 20 females who were Tswana speakers with varying educational levels. All were interviewed on the same day in two groups, first the females and then the males to obtain gender-specific descriptors of LBP severity. The first author was present while a Tswana-speaking research assistant conducted the interviews.

The female participants were each invited to suggest two or more different Tswana descriptors or terms that they felt best described each of the following categories of pain, namely, mild, moderate, severe and worst possible pain. This was done in an open forum and once consensus was reached the suggested descriptors were written on a blackboard. The group then voted for the descriptors for each category by a show of hands. All descriptors that received over 70% of the votes were included in the second phase, one for mild pain, three for moderate, two for severe and one for worst possible pain, ranked and recorded on the blackboard in ascending order. The same process was followed for the male group, who ended up with one descriptor for mild pain, two for moderate, three for severe and one for worst possible pain. Both groups appeared to have difficulty in agreeing on only one description for moderate and one for severe pain.

These sessions were followed by a lunch break and an hour’s rest in separate venues. The male and female descriptors were paired, resulting in seven sets. The combined group of males and females were then asked to vote for one of both the male and female descriptors in each pain category of mild, moderate, severe and worst pain. Seventy per cent (or the highest value if less than 70%) was taken as the cut-off for acceptance of an appropriate descriptor for each category of pain. The final scale thus had the most popular descriptor for each of the four levels of pain for each gender. This process of data collection took a total of 8 hours. The descriptors were then translated into English using the translation process described by Beaton, Bombardier, and Guillemin (2000) and Ostlund, Gustavsson and Furst (2006). This scale was then named the TVPDS.

### Part 2: Testing the scale in patients with low back pain

Two hundred and fifty participants with LBP, who were either packers from four supermarket groups or patients at clinics for LBP in the same South African province where Part 1 was conducted, were approached and asked to participate in Part 2 of the study. The inclusion criteria were the same as for Part 1. The sample size was calculated according to Jenson, Turner and Romano (1994), who suggest that 10 participants per scale item are required to test a scale (Jensen et al. 1994). Only people with LBP were included but there was no assessment regarding details of the pain.

Participants were asked to provide basic demographic data on age, gender and level of education and then to rate their present pain (PP) perception out of 10 (nought being ‘no pain’ and 10 ‘worst possible pain’) and this was recorded. They then selected their perceived level of PP on the TVPDS, a numerical visual analogue scale (NVAS) (in integers between 0 and 10 with the numbers below a 10 cm line), the Pakistani Coin Heap Scale on a 10 cm line (modified from Salim 1993 to have South African rand coins) and the Wong–Baker FACES pain rating scale (WBFPS) (Salim 1993; Wong & Baker 1988). The TVPDS in Tswana, NVAS, Pakistani Coin Heap Scale and WBFPS are presented in Appendix 1.

The median PP intensity was calculated for each of the four categories of the TVPDS. These medians were then used to compare to the other scales. The medians of the six faces of the WBFPS were taken as 0, 1.6, 3.7, 5.7, 7.4 and 9.5 as described by Garra et al. (2010). Pain level on the coin scale was measured from the right anchor and described in centimetres (Salim 1993).

Age was described using means and standard deviations and comparisons were done with an unpaired *t*-test. Percentages were used to describe categorical data. All pain data were described using medians and ranges. Non-parametric analysis was used for all comparisons. Thus, Spearman correlation, Mann–Whitney test and Kruskal–Wallis ANOVA with Dunn’s post-hoc test were used for numerical data and Fisher’s exact test was used for comparison of categorical data. Wilcoxon matched pairs test was used to compare PP data with TVPDS data.

### Ethical consideration

All participants signed informed consent forms and institutional ethical clearance was obtained from the University of the Witwatersrand (M091121). Permission was obtained from the hospital, clinics and various businesses involved in the study.

## Results

### Part 1: Development of the scale

The mean age of the group of 40 participants was 44 years of age, and there was no significant difference between the group of males (mean [SD] = 44.4 [11.7]) and the group of females (mean [SD] = 44.1 [10.3], *p* = 0.932 unpaired *t*-test). Both groups had a wide range of educational levels.

The most common original verbal pain descriptors developed by both genders to describe varying degrees of severity of pain, as translated into English, are indicated in Table 1, as well as the percentage of the gender that agreed to that description for both rounds of voting. All data presented on phrase choice indicate the English version of the Tswana phrases to allow for easy reading.

**TABLE 1:** English phrases used to describe four levels of severity in low back pain in Tswana-speaking patients for both genders.

Levels	Male	Round 1 % (95% CI)	Round 2 % (95% CI)	Female	Round 1 % (95% CI)	Round 2 % (95% CI)
Mild	**Pressing on a bruise**	75 (58.5–86.8)	85 (69.5–93.8)	**Pricked by a pin**	80 (63.9–90.4)	80 (63.9–90.4)
Moderate	**Stubbing your toe**	75 (58.5–86.8)	75 (58.5–86.8)	Scratched by a cat or branch	72 (55.8–84.9)	-
	Bee sting	72 (55.8–84.9)	-	**Bumping your elbow**	80 (63.9–90.4)	70 (53.3–82.9)
	-	-	-	Toothache	78 (61.1–88.6)	-
Severe	Something heavy falling on foot	75 (58.5–86.8)	-	A fall on your bottom	70 (53.3–82.9)	-
	**Hammer blow on finger**	80 (63.9–90.4)	75 (58.5–86.8)	**Cut by a knife**	80 (63.9–90.4)	75 (58.5–86.8)
	A stab in your back	80 (63.9–90.4)	-	-	-	-
Worst	**Burnt by fire**	93 (78.5–98)	95 (81.8–99.1)	**Burnt by boiling water**	90 (75.4–96.8)	100 (89.1–100)

Note: Round 1 represents the first round of voting, where males and females were in separate groups. Round 2 represents the votes cast by each gender for their own items in the second round for the final shortened version of the scale. Those phrases selected for the final version are indicated in bold. Numbers in brackets indicate 95% confidence intervals.

After selection of the final items the votes for the phrases for mild and worst pain either stayed the same or improved, while the phrases for moderate and severe pain had a loss of votes. Less than 30% of the opposite gender voted for any particular item during the second round of voting.

### Part 2: Testing the scale in patients with low back pain

The distribution of pain scores for PP in the 250 participants with LBP is indicated in Figure 1. There was a good spread of pain perception across the participants.

**FIGURE 1:**
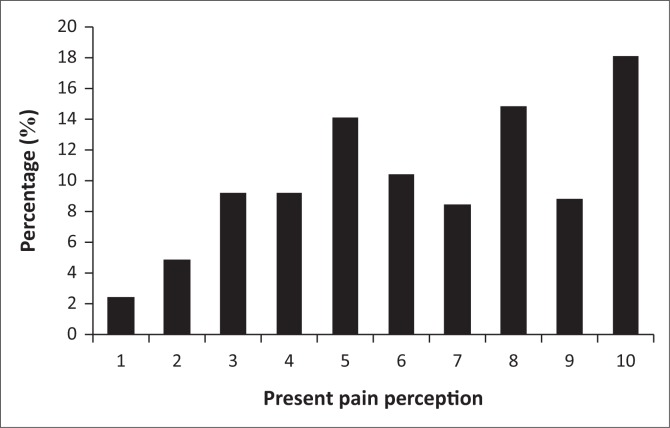
Distribution of present pain perception of participants.

The median present PP for each of the categories of the TVPDS is indicated in Figure 2. The median confidence intervals (CI) of PP for each category of the TVPDS were as follows: mild, 4 (3.51–4.49); moderate, 5 (5.0–5.0); severe, 8 (7.52–8.58); and worst, 10 (9.51–10.0), indicating a non-linear distribution. The medians of all groups were significantly different from each other (*p* < 0.0001: Kruskal–Wallis ANOVA) except for the comparison between mild and moderate pain, which was not significantly different (*p* > 0.05).

**FIGURE 2:**
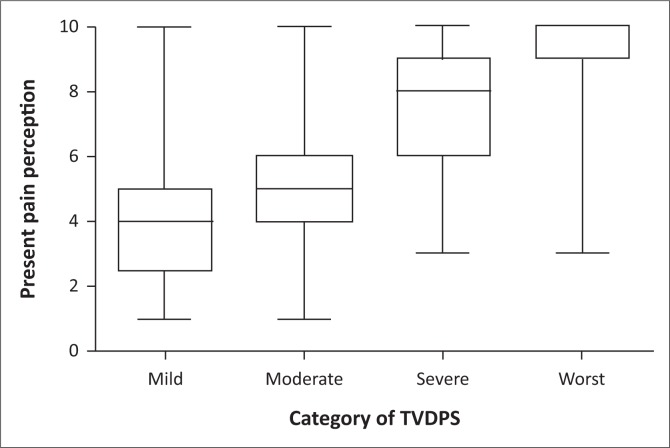
Categories of Tswana Verbal Pain Descriptor Scale (TVPDS).

The correlation matrix analysis (*R* values) between the four different scales indicated that the correlations were all moderate to high (*p* < 0.0001) (Table 2).

**TABLE 2:** A correlation matrix analysis of the four pain scales.

Variable	NVAS	Wong–Baker FACES Scale	Pakistani Coin Heap
TVPDS	0.704	0.695	0.717
NVAS	-	0.779	0.880
Wong–Baker FACES scale	-	-	0.864

Note: Correlations were all moderate to high (*p* < 0.0001).

TVPDS, Tswana Verbal Pain Descriptor Scale; NVAS, numerical visual analogue scale.

When comparing females and males on their PP perception the 178 females had a significantly higher level of pain than the 72 males (females 7 [range 1–10] and males 5 [range 1–10] – Mann–Whitney test *p* = 0.0023). This significant difference was maintained on the TVPDS (females 8 [range 4–10] and males 5 [range 4–10] – Mann–Whitney test *p* = 0.0303). The scores on the TVPDS scale were weakly, correlated with age (*r* = 0.153, *p* = 0.016) but not with level of education (*r* = –0.0951, *p* = 0.135 – all non-parametric Spearman correlations).

## Discussion

The TVPDS has the potential to be a useful clinical tool for the measurement of LBP in persons who speak Tswana, of both genders, differing education levels and ages. It was developed by a group of Tswana-speaking individuals with LBP and then tested in a cohort of 250 persons also with LBP, correlating well with their perception of pain, measured on the NVAS. In addition it correlated well with the six-face Wong–Baker FACES Pain Rating Scale and the Pakistani Coin Heap Scale (Garra et al. 2010; Wong & Baker 1988). The development of this scale means that Tswana-speaking patients with LBP now have an incremental verbal descriptor pain scale that has the potential to be used clinically.

The African prevalence of LBP is similar to the rest of the world, with a lifetime prevalence of 62% (Louw, Morris & Grimmer-Somes 2007). LBP is therefore a common health problem managed by health care professionals in a variety of different facilities and in patients from different languages in South Africa (Callister 2003). Health care professionals and patients need to understand one another as they attempt to establish the level and type of pain being experienced. Patients must be able to express their pain in a way that is meaningful to them (Schott 2004; Semino 2010; Stewart 2014) but that is also meaningful to practitioners. There is always the difficulty that patients and health-care practitioners express pain using different descriptions if they are from different cultures and languages (Stewart 2014). Thus this scale provides Tswana-speaking patients with LBP a meaningful way of expressing pain categories that, when translated into English, are understandable to health-care practitioners as well.

The process of developing this scale, we believe, should be used more often. Pain scales are usually developed by health-care practitioners and not by patients (Garra et al. 2010; Wong & Baker 1988). The uniqueness of the development of this scale lies firstly in it being a pain scale using everyday pain descriptions developed solely by a group of patients and secondly in how they were facilitated. The TVPDS was developed from first principles by patients with LBP without using or comparing any established pain scales, which may have influenced their thinking. Similarly the participants were not inhibited by the guidance of a health-care practitioner. The group attended the same clinic; lived in similar socio-economic circumstances; and were from the same cultural and language backgrounds (Wagstaff, Smith & Wood 1985). The facilitator lived in the same area, was Tswana speaking and familiar with the nuances of the language, socio-economic status and culture of the group. This meant that patients were comfortable in expressing themselves in their first language and were given the opportunity to choose and debate everyday phrases that they felt best described their pain (Callister 2003; Schott 2004; Semino 2010; Stewart 2014). Those descriptors finally chosen had at least a 70% agreement among participants.

The abstract concepts of levels of pain severity usually presented in an unfamiliar language were now articulated in the participants’ first language by using descriptions of known everyday situations that cause pain. The use of these everyday descriptions to promote understanding of increasing levels of pain severity by providing concrete examples of abstract ideas is well illustrated here (Jones et al. 2007; Manyani & Mathipa 2014; Wagstaff, et al 1985). The descriptions developed for pain in this study are not specific to a rural population and so could possibly be used across a wider socio-economic spectrum of patients, although this would need further testing (Manyani & Mathipa 2014; Wagstaff et al. 1985).

The everyday descriptions of pain chosen for the scale differed between genders as expected. The females in this study, who were mostly homemakers, associated the categories of pain with their household activities, by using the description ‘like being cut with a knife’ at the mild end of the scale and ‘like being burnt with water’ as the worst possible pain. The males, who worked in a largely farming and mining community, described concepts ‘like being hit with a hammer’ (moderate pain) and ‘like being burnt by fire’ (worst pain) (Wagstaff et al. 1985). Both genders had difficulty in finding one description to describe the differences between mild and moderate pain and did not accept the descriptive phrases used by the other gender. This could either be a reflection of their stoicism as a result of living in poor, difficult circumstances (Yong et al. 2001) or that people involved in the usual manual activities of these communities tend to be more affected by LBP and thus their experiences of pain tend always to be more severe (Punnet 2006). Both these possibilities need further exploration. The significantly greater pain experienced by the female group may suggest the differences in pain perception between genders (Channing et al. 2009). The increasing levels of pain experienced by the older participants are also not unexpected, given the possible increasing levels of pathology (Thomas et al. 2004).

The differences in the number of females versus males in this study illustrate the reality of public health care in South Africa. It is mostly women who attend public health care facilities because the men are likely to be in full-time employment. The educational levels also illustrate the employment categories of people attending public health care facilities (Harris et al. 2011).

The good correlation found in the 250 participants with LBP between their perception of pain rated on a scale of 1–10 and the TVPDS suggests that this scale can be used to distinguish among incremental categories of pain using descriptions that are potentially more meaningful to patients than are numbers, as they describe familiar pain-provoking situations. The moderate to good correlations seen with the TVPDS and both the WBFPS and the Pakistani Coin Heap Scale imply that this scale is equivalent to established pain scales that have been used in similar socio-economic situations (Garra et al. 2010; Wong & Baker 1988). The TVPDS can thus be substituted for scales like these and used in situations where Tswana is the language of choice of patients, demonstrating the usefulness of language to describe levels of pain.

The limitations of the study include the use of only one language of the 11 South African languages and the fact that the results cannot be extrapolated to other language groups. However, the descriptions used by our participants were not specific to this population so testing them in other South African languages should be considered. It is also not clear what, if any, influence the rural nature of the surroundings had on the descriptors used.

## Conclusion

The everyday pain descriptors in this study were developed by patients with LBP. As the descriptors they used are not specific to a particular group of people, the scale could be useful in a variety of different South African clinical situations but needs further translation and testing. The manner in which the scale was developed is unique, being patient driven. The moderate to good correlations found with the NVAS, the WBFPS and the Pakistani Coin Heap Scale suggest that in addition to being able to describe pain in Tswana using everyday pain descriptors, the scale can be used in place of pain scales developed elsewhere.

## Figures and Tables

**FIGURE 1-A1:**
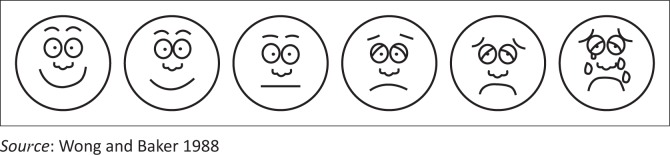
The Wong–Baker FACES Scale.

**FIGURE 2-A1:**
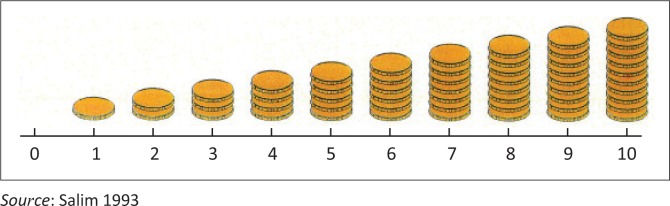
The Pakistani Coin Heap Scale.

**FIGURE 3-A1:**
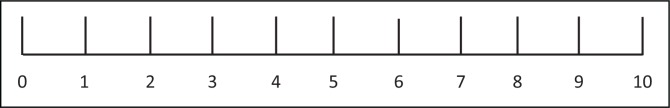
SetlhabiSepe Sethlhabi se se tseneletseng (o thlabiwa ke setlhabi). The numerical visual analogue scale.

## Appendix 1

**TABLE 1-A1:** Tswana version of the Tswana Verbal Pain Descriptor Scale showing male and female descriptors.

Degree of pain	Gender	Tswana phrase	English phrase
Mild	Male	Jaaka go gatelela mo leteketong	Pressing on a bruise
	Female	Jaaka go tlhabiwa ka nelete	Pricked by a pin
Moderate	Male	Jaaka go thula sekgono	Bumping your elbow
	Female	Jaaka go kgopa monwanwa	Stubbing your toe
Severe	Male	Jaaka go ikitla mo monwaneng ka hamole	Hammer blow on finger
	Female	Jaaka go segiwa ka thipa	Cut by a knife
Worst	Male	Jaaka go fisiwa ke molelo	Burnt by fire
	Female	Jaaka go fisiwa ke metsi a a belang	Burnt by boiling water
